# The Nitrate-Nitrite-Nitric Oxide Pathway on Healthy Ageing: A Review of Pre-clinical and Clinical Data on the Impact of Dietary Nitrate in the Elderly

**DOI:** 10.3389/fragi.2021.778467

**Published:** 2021-11-17

**Authors:** Bárbara S. Rocha

**Affiliations:** Faculty of Pharmacy and Center for Neuroscience and Cell Biology, Health Sciences Campus, Azinhaga de Santa Comba, University of Coimbra, Coimbra, Portugal

**Keywords:** nitrate, nitrite, nitric oxide, ageing, diet

## Abstract

We are living longer. Are we living healthier? As we age, cellular and molecular damage reshape our physiological responses towards environmental and endogenous stimuli. The free radical theory of ageing has been proposed long before ageing has been considered a “scientific discipline” and, since then, has been discussed and upgraded as a major contributor to aberrant ageing. Assuming that ageing results merely from the accumulation of oxidative modifications of biomolecules is not only a simplistic and reductive view of such a complex and dynamic process, but also free radicals and related oxidants are now considered pivotal signalling molecules. The fine modulation of critical signalling pathways by redox compounds demands a novel approach to tackle the role of free radicals in ageing. Nitric oxide (^⋅^NO) is a paradigmatic example given its biological functions in cardiovascular, neurologic and immune systems. In addition to the canonical ^⋅^NO synthesis by a family of enzymes, nitrate from green leafy vegetables, is reduced to nitrite in the oral cavity which is further reduced to ^⋅^NO in the stomach. Boosting this nitrate-nitrite-NO pathway has been shown to improve gastrointestinal, cardiovascular, metabolic and cognitive performance both in humans and in animal models of disease. In the elderly, nitrate-derived ^⋅^NO has been shown improve several physiological functions that typically decline during ageing. In this paper, the role of nitrate and derived nitrogen oxides will be discussed while reviewing pre-clinical and clinical data on the cardiovascular, neuronal, musculoskeletal and metabolic effects of nitrate during healthy ageing.

## Introduction: From the Free Radical Theory of Ageing to the Nitrate-Nitrite-Nitric Oxide Pathway

Ageing and the physiological events that limit lifespan have been subject of intense research for many decades. Increasing age is not only associated with physiological alterations in different organs, but is also the major risk factor for the most prevalent diseases of the XXIst century including cardiovascular, oncological, neurodegenerative and metabolic disorders (Niccoli and Partridge, 2012). Ground breaking discoveries on the molecular mechanisms of ageing have unveiled environmental, genetic and intracellular signalling pathways, involving target of rapamycin (TOR) proteins and insulin-like signalling cascades, as potential drivers of age-associated cellular dysfunction (recently reviewed in (Campisi et al., 2019)) and the hallmarks that represent common denominators of mammal aging in different organisms have been proposed (López-Otín et al., 2013). In the 1950s, the overproduction of reactive oxygen species and the accumulation of oxidative modifications, known as the *free radical theory of ageing*, has been proposed as a driver of biological ageing (Harman, 1956). However, the production of reactive oxygen species cannot be envisaged as the sole or even major driver of ageing because these compounds are a chemically and biologically diverse group of molecules derived from molecular oxygen with critical signalling functions under physiological conditions, ensuring what has been called the *oxidative eustress* (Viña et al., 2007; Sies et al., 2017; Borras et al., 2020). Also, data from different research groups have not only shown that antioxidants do not prevent the molecular mechanisms of ageing and age-related disorders (Viña et al., 2018) but also that, from the manipulation of several antioxidant genes, only the deletion of Sod1 gene reduced lifespan (Pérez et al., 2009). Taken together, the free radical theory of ageing is now considered a simplistic and outdated hypothesis. Accordingly, superoxide radical and hydrogen peroxide are two emerging examples of how oxidants may be produced by tightly controlled enzymatic reactions (Sies and Jones, 2020) and, nitric oxide (^•^NO) is an additional example of a pleiotropic signalling radical with physiological relevance (Moncada and Higgs, 2006).

Nitric oxide is a small, hydrophobic gas that freely permeates biological membranes and interacts with molecular targets within its diffusional spread, ensuring physiological functions such as vasodilation, innate immune response and neuromodulation (Rocha et al., 2011; Ledo et al., 2005; Moncada and Higgs, 1993). Along with the canonical L-arginine-NO pathway (Moncada and Higgs, 1993), ^•^NO is also produced from nitrate through the nitrate-nitrite-NO pathway (Lundberg et al., 2008). The latter is particularly relevant since ^•^NO is produced from nitrate, traditionally regarded as an end product of ^•^NO oxidation, without the involvement of NO synthases (NOS) (Benjamin et al., 1994; Lundberg et al., 1994). Also, the major source of nitrate are green leafy vegetables and roots such as lettuce, spinach, rucola and beetroot (Weitzberg and Lundberg, 2013). Thus, the case can be made that the synthesis of a pleiotropic signalling molecule may directly depend on human dietary behaviour and, in fact, nitrate is now recognised as the most significant biological precursor of ^•^NO *in vivo* (Lundberg et al., 2008). As we chew, green vegetables release nitrate to saliva which is swallowed and absorbed in the small intestine. About 25% of circulating nitrate is transported by sialin, an electrogenic nitrate/H^+^ transporter, into the salivary glands and secreted into the oral cavity (Lundberg et al., 2008; Qin et al., 2012). This enterosalivary circulation supplies nitrate to the oral microbiota that uses nitrogen to produce ATP while reducing nitrate to nitrite (Fritsch et al., 1985). Once swallowed, nitrite is reduced to ^•^NO and other bioactive reactive nitrogen oxides in the stomach (Lundberg et al., 2009; Rocha et al., 2012). This is a chemical rather than enzymatic reaction, as nitrite is protonated to nitrous acid which decomposes to ^•^NO and other oxidants (Benjamin et al., 1994). However, most nitrite is absorbed into the bloodstream, triggering ^•^NO-dependent and independent signalling pathways in every organ system (Lundberg and Weitzberg, 2005; Bryan et al., 2007; Rocha et al., 2016; Lundberg et al., 2018). Nitrate-derived ^•^NO has been shown to increase gastric mucosal blood flow and mucus production, to eradicate gut pathogens and prevent inflammatory events associated with peptic ulcer disease (Benjamin et al., 1994; Dykhuizen et al., 1998; Björne et al., 2004; Jädert et al., 2012; Rocha et al., 2013). Systemically, nitrate was shown to serve as a *reservoir* of ^•^NO under hypoxic conditions as several enzymes acquire a nitrite-reductase activity under these conditions (van Faassen et al., 2009). By increasing the bioavailability of ^•^NO and nitroso derivatives, nitrate prevents not only vascular inflammatory events and atherogenesis, but also platelet aggregation and myocardial ischemia-reperfusion injury (Webb et al., 2004; Lundberg and Weitzberg, 2005; Shiva and Gladwin, 2009). Also, by increasing the expression of mitochondrial respiratory complexes and the synthesis of anti-inflammatory cytokines, nitrate prevents visceral fat accumulation and hyperglycaemia associated with metabolic syndrome (Carlström et al., 2010; Lundberg et al., 2018). While these metabolic effects have been shown to be associated with the inhibition of NADPH oxidase (Hezel et al., 2016), other molecular targets have also been described, such as AMP-activated protein kinase (AMPK) (Cordero-Herrera et al., 2019).

In this short review, it will be discussed pre-clinical and clinical data on the impact of nitrate in cardiovascular, metabolic, musculoskeletal and neurological diseases in the elderly. The mitigation of age-associated co-morbidities by nitrate will also be discussed and the translational opportunities of this anion will be highlighted.

## The Impact of Dietary Nitrate on Age-Related Co-morbidities

**FIGURE 1:**
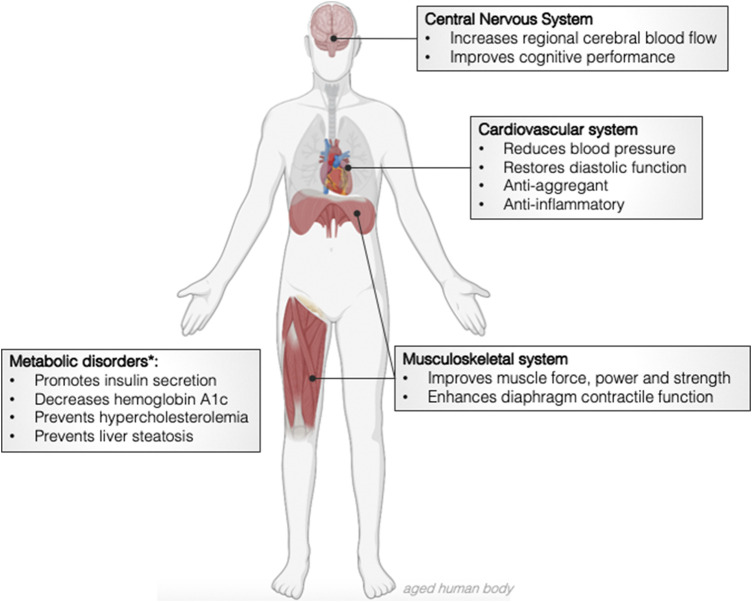
Effects of dietary nitrate in the physiological events associated with healthy ageing and age-related disorders. *pre-clinical and clinical data obtained from middle age and old volunteers.

The demonstration that nitrate is reduced to nitrite and ^**•**^NO in the gut, yielding up to 40,000 ppb, changed the paradigm of redox biology (Gago et al., 2007; Rocha et al., 2009). Never such high steady state concentrations of ^**•**^NO have been reported *in vivo* and, given the acidic pH of the stomach, the chemical complexity leading to the production of other oxidants (nitrogen dioxide radical, peroxynitrite, dinitrogen trioxide) was (and still is) largely unclear in a biological setting (Rocha et al., 2012; Lundberg and Weitzberg, 2013). The impact of such fluxes of ^**•**^NO arising from the gastric milieu was soon associated with gastrointestinal effects. Nitrate-derived ^**•**^NO was shown to diffuse towards the gastric mucosa inducing local vasodilation (Björne et al., 2004; Rocha et al., 2010), the expression of genes encoding mucins, the glycoproteins that sustain the gastric mucus, and to inhibit inflammatory pathways such as those involving myeloperoxidase and the expression of adhesion molecules such as ICAM and P-selectin (Jädert et al., 2012; Peleli et al., 2019). Such anti-inflammatory properties have been shown to prevent peptic ulcer disease both induced by NSAIDs or not and alleviate histological and clinical signs of inflammatory bowel disease (Jansson et al., 2007; Borniquel et al., 2010; Rocha et al., 2013). The production of ^**•**^NO-derived compounds, more stable than ^**•**^NO itself, such as nitroso compounds and nitroalkenes, that can be absorbed into the systemic circulation, opened new avenues on the putative systemic effects of nitrate (Bonacci et al., 2012; Kelley et al., 2014; Delmastro-Greenwood et al., 2015). Also, after a meal containing nitrate, plasma nitrate and nitrite increase in approximately 30 min and remain high for 5–6 h due to the enterosalivary circulation of nitrate (Lundberg and Govoni, 2004). Hence, regarding the systemic effects of nitrate, one needs to consider both the physiologically active molecules that are produced and absorbed from the gut and the signalling events elicited by circulating nitrite. Although plasma nitrite increases typically from 120 nM under fasting to 400 nM after an oral nitrate load (10 mg/kg) (Lundberg and Govoni, 2004), this is sufficient to produce ^**•**^NO under hypoxia. At this pO_2_, several enzymes, including haemoglobin, myoglobin and xanthine oxidase acquire a nitrite reductase activity, reducing nitrite to ^**•**^NO (for a comprehensive review see (van Faassen et al., 2009)). Also, under hypoxia, and since oxygen is a co-factor for NOS, the activity of these enzymes is inhibited and nitrite reduction is the only source of ^**•**^NO at locations where vasodilation is mandatory to prevent or revert the effects of oxygen privation. These observations from the past 2 decades prompted several pre-clinical and clinical studies with the aim of using nitrate to prevent a wide range of diseases (Rammos et al., 2016; Bettio et al., 2017; Raubenheimer et al., 2017; Coggan and Peterson, 2018). Curiously, many of these disorders are frequent co-morbidities in the elderly and deviate not only what would be a healthy decay of organ functions, but also compromise the quality of life and ultimately, lifespan (Niccoli and Partridge, 2012; Divo et al., 2014). During healthy ageing, several anatomical and functional alterations occur in all organ systems. To cite just a few examples, there is a 1) decline of muscle and bone mass, 2) reduction of the functional capacity of neurons, 3) decrease of gastrointestinal motility and gastric acid production, 4) reduction of renal weight and size due to the loss of glomeruli and 5) increase of the anteroposterior diameter of the thorax in addition to an increased thoracic rigidity (Sharma and Goodwin, 2006). Accumulating evidence suggest that nitrate may prevent or mitigate these age-related alterations and promote healthspan, the healthy life expectancy. This data will now be discussed and is summarised in Figure 1.

## Cardiovascular System

It has been demonstrated that dietary nitrate is associated with improved cardiovascular health (Bryan et al., 2007; Borlaug et al., 2015; Kapil et al., 2015; Hezel et al., 2016). Indeed, given that green leafy vegetables are the major source of nitrate, and that the consumption of such foods is recommended by the World Health Organisation to prevent major cardiovascular events (Appel et al., 1997), it is not surprising that nitrate supplementation has been investigated as a therapeutic strategy to reduce cardiovascular mortality and morbidity (Hung et al., 2004; Carter et al., 2010; Goh et al., 2019). An increase of systolic blood pressure is one of the physiological hallmarks of ageing due to increased arterial stiffness and left ventricle afterload with consequent ventricular hypertrophy (Cheitlin, 2003). Hence, the impact of nitrate on blood pressure has been extensively studied both in young and old adults as well as in normotensive and hypertensive volunteers (Larsen et al., 2006; Gilchrist et al., 2011; Kapil et al., 2015). Data suggests that the stimulation of the nitrate-nitrite-NO pathway reduces arterial blood pressure. Depending on the experimental design (acute or sub-acute nitrate administration) and the form of nitrate intake (beetroot juice or sodium nitrate), dietary concentrations of nitrate have been shown to reduce diastolic blood pressure by 3.7 mmHg (Larsen et al., 2006) or both diastolic (8.1 mmHg) and systolic (4.4 mmHg) blood pressure (Webb et al., 2008). In older adults, one needs to consider age-associated changes on oral microbiome and a reduced salivary rate (Percival et al., 1991) that may prevent the blood pressure lowering effects of nitrate. Nevertheless, Vanhatalo and co-workers have elegantly shown that a 10-days supplementation of nitrate increases plasma nitrite while reducing both systolic and mean arterial pressure in normotensive old volunteers (age range 70–79 years) (Vanhatalo et al., 2018). Nitrate supplementation also altered the composition of the oral microbiome, increasing the relative abundance of *Rothia* and *Neisseria* and decreasing Prevotella and Veillonella, which correlated with a higher increase in plasma nitrite (Vanhatalo et al., 2018). Similarly, an acute nitrate load, was also shown to decrease both diastolic and systolic blood pressure by approximately six and 7.5 mmHg, respectively, in a group of old volunteers (age range 50–70 years) (Stanaway et al., 2019). The increase of plasma nitrite was also significantly higher in old rather than young subjects (Stanaway et al., 2019), suggesting that boosting the nitrate-nitrite-NO pathway may have an unexpected better outcome in terms of cardiovascular indicators in the elderly. Nevertheless, ageing is not only associated with a vasoconstrictive state but also with pro-coagulant and pro-inflammatory events (Cheitlin, 2003). In this regard, an additional study has replicated both the systolic and diastolic blood pressure lowering effects of nitrate in healthy older adults (mean age 64), but has also shown a reduction in CD11b-expressing granulocytes as well as in blood monocyte-platelet aggregates, suggesting a novel anti-adhesive phenotype (Raubenheimer et al., 2017). Finally, diastolic dysfunction, with impaired passive filling, leads to heart failure, a cardiac disease with high prevalence among elderly populations (Wan et al., 2014). In aged mice, chronic nitrate supplementation accelerates cardiomyocyte calcium handling by increasing LTCC flux, a L-type calcium channel that controls sarcoplasmic reticulum calcium release (Rammos et al., 2016). Also, nitrate was shown to promote ^**•**^NO-cGMP-PKG signalling and to increase the levels of cardiac nitrosothiols while reversing age-related diastolic dysfunction and improving vascular function (Rammos et al., 2016).

By promoting systemic ^•^NO synthesis, nitrate has been shown to inhibit the vasoconstrictive, pro-adhesive and pro-coagulant tendency observed with ageing. Thus, nitrate supplementation may be hypothesized in the field of gerontology to prevent age-associated cardiovascular morbidities.

## Central Nervous System

Cognitive impairment, working memory decline and poor executive functioning are the most frequent neurological deficits during healthy ageing (Bettio et al., 2017). Chronic inflammation and cerebral hypoperfusion are fundamental contributors for the decay of cognition and executive functions (Ruitenberg et al., 2005). Anatomical and functional alterations of cerebral blood vessels, including increased tortuosity and diminished ^•^NO bioavailability, contribute to a chronic ischemic environment in the aged brain (Moody et al., 1995). Hence, it has been hypothesised that nitrate could be reduced to nitrite by the oral microbiota and, in turn, nitrite would be univalently reduced to ^•^NO in the cerebral parenchyma either chemically or by specific enzymes that acquire a nitrite reductase activity at low pO_2_ (Millar, 1995; van Faassen et al., 2009). This could be interpreted as a dietary approach to increase ^•^NO bioavailability in the brain of older adults and, in fact, Presley et al. have demonstrated that a diet rich in nitrate increases cerebral blood flow in old human volunteers (mean age 75) (Presley et al., 2011). Curiously, dietary nitrate does not increase global cerebral blood flow but rather induces vasodilation in the dorsolateral prefrontal cortex, a region responsible for higher executive functions (Presley et al., 2011). The vasodilatory effect of nitrate, upon reduction to nitrite and ^•^NO, was also observed in the prefrontal cortex of young adults and was associated with improved cognitive performance (Wightman et al., 2015). Curiously, blood flow diminishes during the least demanding cognitive tasks (Wightman et al., 2015), suggesting that nitrate-dependent vasodilation affords an additional backup of nutrients and oxygen needed to accomplish complex cognitive tasks. However, other studies did not replicate the improvement of cognitive performance in older adults (Kelly et al., 2013). In a study by Kelly et al., old volunteers (mean age 63—females; 64—males), showed no improvement neither in serial subtractions, rapid information processing nor number recall tasks (Kelly et al., 2013). One possible explanation is that these volunteers were about 10 years younger than the study populations included in other studies which may mask the effect of nitrate since cognition may still be quite well preserved. Also, while in other studies (Presley et al., 2011), volunteers were exposed to a nitrate-rich diet (a list of foods high in nitrate was provided to participants), in this case volunteers were exposed to a higher dose of nitrate (24.6 mmol for 2.5 days) (Kelly et al., 2013).

Taken altogether, despite the vasodilatory effect of nitrate-derived ^•^NO in the aged brain, additional studies are necessary to ascertain the impact of the nitrate-nitrite-NO pathway in cognitive performance and memory processing in the elderly.

## Musculoskeletal System

During healthy ageing, there is a predictable decline in skeletal muscle force, speed and strength that may limit or even disable the accomplishment of daily life activities (Roshanravan et al., 2017). Dietary nitrate, through the chemical reduction to nitrite and ^•^NO in the gut, has been shown to enhance the contractile function of the skeletal muscle not only in young but also in old human volunteers (Haider and Folland, 2014; Coggan et al., 2017). In old adults (mean age 71), acute nitrate supplementation increases plasma nitrate, nitrite and exhaled ^•^NO while improving knee extensor power and angular velocity, paralleling similar observations in young adults (Coggan et al., 2020). In this group age, nitrate increases both evoked muscle force production as well as excitation-contraction coupling of the skeletal muscle (Haider and Folland, 2014). The molecular mechanisms underlying such effects include an increase of ^•^NO bioavailability and the downstream phosphorylation of myofibrillar proteins (Coggan and Peterson, 2018). In addition, nitrate has also been shown to promote muscle force production *in vitro* by increasing the expression of calcium handling proteins thereby improving intracellular calcium handling (Hernández et al., 2012). These observations suggest that nitrate supplementation in the elderly may preserve motility, independence and even prevent premature death. Recently, Kumar and co-workers have also shown that nitrate improves diaphragmatic contractile function in old rodents (Kumar et al., 2020), suggesting that the ventilatory movements may be facilitated in old subjects complying with a nitrate-rich diet. In addition to the anatomical changes of the thorax with ageing and the loss of elastin, the contractile function of the diagram also declines with age, leading to an insufficient airway clearance and breathing complications which may ultimately increase the risk of pulmonary infections (Kelley and Ferreira, 2017). A physiological dose of nitrate increases the rate of force development and peak power of the diaphragm of old mice but without affecting the phosphorylation status of myofibrillar proteins or the abundance of calcium handling proteins (Kumar et al., 2020). Finally, the molecular mechanisms underlying the ergogenic effects of nitrate are also associated with an increase of mitochondrial bioenergetics (Jones et al., 2018) as nitrate improves oxidative phosphorylation efficiency (P/O ratio) while reducing oxygen cost during exercise in young (mean age 25 ± 1 year) human volunteers (Larsen et al., 2011). From a mechanistic viewpoint, nitrate has been shown to inhibit the expression of ATP/ADP translocase, to increase the efficiency of ATP synthesis and of ATP-consuming metabolic pathways (Larsen et al., 2011; Affourtit et al., 2015). Also, nitrite derived from nitrate, induces posttranslational modifications of mitochondrial respiratory complexes (such as S-nitrosation of complex I) dampening electron transfer, which may have a particular interest during ischemia/reperfusion events (Shiva 2007) (Shiva et al., 2007). Of note, most of the studies regarding mitochondrial bioenergetics were performed in young volunteers and therefore robust data on old populations are necessary to a acknowledge the impact of nitrate on mitochondrial pathways in the elderly. Although the molecular mechanisms remain unclear, it is now apparent that nitrate, by increasing the bioavailability of ^•^NO, preserve the contractile function of the skeletal muscle, likely promoting independent routines and an improved quality of life.

## Metabolic Disorders

The impact of dietary nitrate on human metabolic pathways has been recently unveiled and include antidiabetic effects as well as the reversal of hallmark features of metabolic syndrome (for a recent review see (Lundberg et al., 2018)). In murine models of metabolic diseases and diabetes, nitrate has been shown to increase insulin secretion and glucose tolerance, reduce haemoglobin A1c, dyslipidemia, visceral fat accumulation and liver steatosis (Stokes et al., 2009; Carlström et al., 2010; Velmurugan et al., 2016). The molecular mechanisms underlying such effects include not only the post-translational modification of mitochondrial respiratory complexes, such as S-nitrosation of complex I and binding to cytochrome c, but also the synthesis of cytokines with anti-inflammatory properties (Lundberg et al., 2018). Also, nitrate-derived nitrogen oxides downregulate NADPH oxidase activity thereby inhibiting the synthesis of superoxide radical and higher oxidants such as peroxynitrite anion (Cordero-Herrera et al., 2019). Regarding the cellular energetic status, nitrate has been shown to activate AMPK, reducing the synthesis of fatty acids, promoting fatty acid oxidation and glucose uptake (Peleli et al., 2015; Cordero-Herrera et al., 2019). These recent observations were made mostly in animal models of disease or in adult populations (mean age 53) (Hezel et al., 2016; Velmurugan et al., 2016; Cordero-Herrera et al., 2019) and thus the metabolic effects of nitrate in older adults remains largely unknown. This is a gap that needs to be rapidly filled since many of these disorders are particularly prevalent in the elderly (Niccoli and Partridge, 2012). Indeed, reports are now emerging suggesting that, in ageing mice, daily nitrate intake prevents hepatic senescence-related dysfunction by decreasing the release of alanine aminotransferase and aspartate aminotransferase as well as intracellular lipid deposition (Wang et al., 2018). Additionally, recent metabolomic analysis have shown that nitrate alters the plasma concentration of small metabolites in healthy older adults and these changes correlate with improved motor, vascular and cognitive function (DeVan et al., 2016; Justice et al., 2015; Johnson et al., 2017). The proof-of-concept provided by pre-clinical and clinical data on the metabolic effects of nitrate in old subjects, should now prompt researchers, clinicians and gerontologists to perform larger clinical trials to ascertain whether nitrate should be used as a supplement to prevent or reverse some of the most prevalent ageing disorders.

## Conclusion and Future Directions

The pre-clinical and clinical data herein summarised suggest that diets rich in nitrate may prevent, reverse or mitigate the physiological decay observed during healthy ageing or age-associated disorders. Cardiovascular dysfunction, neurological deficits and metabolic impairment are the major causes of morbidity and mortality among older adults but nitrate supplementation is now emerging as a nutritional approach to enhance cognitive and functional abilities in the elderly. However, some questions remain to be answered. For instance, given that nitrate improves muscle mitochondrial function and oxygen consumption in young volunteers, how would it impact in muscle mitochondria in older adults? Would changes in ^•^NO bioavailability in old subjects interfere with the mitochondrial effects of nitrate observed in young populations? May mitochondrial function and intracellular calcium handling concur to improve muscle contractile function in old subjects? Finally, given the prevalence of metabolic disorders in the elderly, it is mandatory to translate the antidiabetic and antilipemic effects of nitrate observed either in rodents or young volunteers to old populations. Thus, the impact of nitrate in the healthy life expectancy should now be studied in large multicentre trials to ascertain if nitrate-rich diets or supplements could be used in the more generalised context of gerontology.

## References

[B1] Affourtit C. Bailey S. J. Jones A. M. Smallwood M. J. Winyard P. G. (2015). On the Mechanism by Which Dietary Nitrate Improves Human Skeletal Muscle Function. Front. Physiol. 6, 211. 10.3389/fphys.2015.00211 26283970 PMC4518145

[B2] Appel L. J. Moore T. J. Obarzanek E. Vollmer W. M. Svetkey L. P. Sacks F. M. (1997). A Clinical Trial of the Effects of Dietary Patterns on Blood Pressure. N. Engl. J. Med. 336 (16), 1117–1124. 10.1056/nejm199704173361601 9099655

[B3] Benjamin N. O'Driscoll F. Dougall H. Duncan C. Smith L. Golden M. (1994). Stomach NO Synthesis. Nature 368 (6471), 502. 10.1038/368502a0 8139683

[B4] Bettio L. E. B. Rajendran L. Gil-Mohapel J. (2017). The Effects of Aging in the hippocampus and Cognitive Decline. Neurosci. Biobehav. Rev. 79, 66–86. 10.1016/j.neubiorev.2017.04.030 28476525

[B5] Björne H. Petersson J. Phillipson M. Weitzberg E. Holm L. Lundberg J. O. (2004). Nitrite in Saliva Increases Gastric Mucosal Blood Flow and Mucus Thickness. J. Clin. Invest. 113 (1), 106–114. 10.1172/jci19019 14702114 PMC300767

[B6] Bonacci G. Baker P. R. S. Salvatore S. R. Shores D. Khoo N. K. H. Koenitzer J. R. (2012). Conjugated Linoleic Acid Is a Preferential Substrate for Fatty Acid Nitration. J. Biol. Chem. 287 (53), 44071–44082. 10.1074/jbc.m112.401356 23144452 PMC3531723

[B7] Borlaug B. A. Koepp K. E. Melenovsky V. (2015). Sodium Nitrite Improves Exercise Hemodynamics and Ventricular Performance in Heart Failure with Preserved Ejection Fraction. J. Am. Coll. Cardiol. 66 (15), 1672–1682. 10.1016/j.jacc.2015.07.067 26449137

[B8] Borniquel S. Jansson E. A. Cole M. P. Freeman B. A. Lundberg J. O. (2010). Nitrated Oleic Acid Up-Regulates PPARγ and Attenuates Experimental Inflammatory Bowel Disease. Free Radic. Biol. Med. 48 (4), 499–505. 10.1016/j.freeradbiomed.2009.11.014 19932165 PMC3290869

[B9] Borras C. Mas-Bargues C. Sanz-Ros J. Román-Domínguez A. Gimeno-Mallench L. Inglés M. (2020). Extracellular Vesicles and Redox Modulation in Aging. Free Radic. Biol. Med. 149, 44–50. 10.1016/j.freeradbiomed.2019.11.032 31783096

[B10] Bryan N. S. Calvert J. W. Elrod J. W. Gundewar S. Ji S. Y. Lefer D. J. (2007). Dietary Nitrite Supplementation Protects against Myocardial Ischemia-Reperfusion Injury. Proc. Natl. Acad. Sci. 104 (48), 19144–19149. 10.1073/pnas.0706579104 18025468 PMC2141922

[B11] Campisi J. Kapahi P. Lithgow G. J. Melov S. Newman J. C. Verdin E. (2019). From Discoveries in Ageing Research to Therapeutics for Healthy Ageing. Nature 571 (7764), 183–192. 10.1038/s41586-019-1365-2 31292558 PMC7205183

[B12] Carlström M. Larsen F. J. Nyström T. Hezel M. Borniquel S. Weitzberg E. (2010). Dietary Inorganic Nitrate Reverses Features of Metabolic Syndrome in Endothelial Nitric Oxide Synthase-Deficient Mice. Proc. Natl. Acad. Sci. USA 107 (41), 17716–17720. 10.1073/pnas.1008872107 20876122 PMC2955084

[B13] Carter P. Gray L. J. Troughton J. Khunti K. Davies M. J. (2010). Fruit and Vegetable Intake and Incidence of Type 2 Diabetes Mellitus: Systematic Review and Meta-Analysis. BMJ 341, c4229. 10.1136/bmj.c4229 20724400 PMC2924474

[B14] Cheitlin M. D. (2003). Cardiovascular Physiology-Changes with Aging. Am. J. Geriatr. Cardiol. 12 (1), 9–13. 10.1111/j.1076-7460.2003.01751.x 12502909

[B15] Coggan A. R. Peterson L. R. (2018). Dietary Nitrate Enhances the Contractile Properties of Human Skeletal Muscle. Exerc. Sport Sci. Rev. 46 (4), 254–261. 10.1249/jes.0000000000000167 30001275 PMC6138552

[B16] Coggan A. R. Leibowitz J. L. Mikhalkova D. Thies D. Broadstreet S. R. Waller S. (2017). Dietary Nitrate and Muscle Power with Aging. Med. Sci. Sports Exerc. 49 (S), 816. 10.1249/01.mss.0000520002.38690.76

[B17] Coggan A. R. Hoffman R. L. Gray D. A. Moorthi R. N. Thomas D. P. Leibowitz J. L. (2020). A Single Dose of Dietary Nitrate Increases Maximal Knee Extensor Angular Velocity and Power in Healthy Older Men and Women. J. Gerontol. A. Biol. Sci. Med. Sci. 75 (6), 1154–1160. 10.1093/gerona/glz156 31231758 PMC7243590

[B18] Cordero-Herrera I. Kozyra M. Zhuge Z. McCann Haworth S. Moretti C. Peleli M. (2019). AMP-activated Protein Kinase Activation and NADPH Oxidase Inhibition by Inorganic Nitrate and Nitrite Prevent Liver Steatosis. Proc. Natl. Acad. Sci. USA 116 (1), 217–226. 10.1073/pnas.1809406115 30559212 PMC6320503

[B19] Delmastro-Greenwood M. Hughan K. S. Vitturi D. A. Salvatore S. R. Grimes G. Potti G. (2015). Nitrite and Nitrate-dependent Generation of Anti-Inflammatory Fatty Acid Nitroalkenes. Free Radic. Biol. Med. 89, 333–341. 10.1016/j.freeradbiomed.2015.07.149 26385079 PMC4684780

[B20] DeVan A. E. Johnson L. C. Brooks F. A. Evans T. D. Justice J. N. Cruickshank-Quinn C. (2016). Effects of Sodium Nitrite Supplementation on Vascular Function and Related Small Metabolite Signatures in Middle-Aged and Older Adults. J. Appl. Physiol. (1985) 120 (4), 416–425. 10.1152/japplphysiol.00879.2015 26607249 PMC4754621

[B21] Divo M. J. Martinez C. H. Mannino D. M. (2014). Ageing and the Epidemiology of Multimorbidity. Eur. Respir. J. 44 (4), 1055–1068. 10.1183/09031936.00059814 25142482 PMC4918092

[B22] Dykhuizen R. S. Fraser A. McKenzie H. Golden M. Leifert C. Benjamin N. (1998). *Helicobacter pylori* Is Killed by Nitrite under Acidic Conditions. Gut 42 (3), 334–337. 10.1136/gut.42.3.334 9577337 PMC1727019

[B23] Fritsch P. de Saint Blanquat G. Klein D. (1985). Excretion of Nitrates and Nitrites in Saliva and Bile in the Dog. Food Chem. Toxicol. 23 (7), 655–659. 10.1016/0278-6915(85)90153-x 4029832

[B24] Gago B. Lundberg J. O. Barbosa R. M. Laranjinha J. (2007). Red Wine-dependent Reduction of Nitrite to Nitric Oxide in the Stomach. Free Radic. Biol. Med. 43 (9), 1233–1242. 10.1016/j.freeradbiomed.2007.06.007 17893036

[B25] Gilchrist M. Shore A. C. Benjamin N. (2011). Inorganic Nitrate and Nitrite and Control of Blood Pressure. Cardiovasc. Res. 89 (3), 492–498. 10.1093/cvr/cvq309 20884639

[B26] Goh C. E. Trinh P. Colombo P. C. Genkinger J. M. Mathema B. Uhlemann A. C. (2019). Association between Nitrate-Reducing Oral Bacteria and Cardiometabolic Outcomes: Results from ORIGINS. J. Am. Heart Assoc. 8 (23), e013324. 10.1161/JAHA.119.013324 31766976 PMC6912959

[B27] Haider G. Folland J. P. (2014). Nitrate Supplementation Enhances the Contractile Properties of Human Skeletal Muscle. Med. Sci. Sports Exerc. 46 (12), 2234–2243. 10.1249/mss.0000000000000351 24681572

[B28] Harman D. (1956). Aging: a Theory Based on Free Radical and Radiation Chemistry. J. Gerontol. 11 (3), 298–300. 10.1093/geronj/11.3.298 13332224

[B29] Hernández A. Schiffer T. A. Ivarsson N. Cheng A. J. Bruton J. D. Lundberg J. O. (2012). Dietary Nitrate Increases Tetanic [Ca2+]iand Contractile Force in Mouse Fast-Twitch Muscle. J. Physiol. 590 (15), 3575–3583. 10.1113/jphysiol.2012.232777 22687611 PMC3547271

[B30] Hezel M. Peleli M. Liu M. Zollbrecht C. Jensen B. L. Checa A. (2016). Dietary Nitrate Improves Age-Related Hypertension and Metabolic Abnormalities in Rats via Modulation of Angiotensin II Receptor Signaling and Inhibition of Superoxide Generation. Free Radic. Biol. Med. 99, 87–98. 10.1016/j.freeradbiomed.2016.07.025 27474450

[B31] Hung H.-C. Joshipura K. J. Jiang R. Hu F. B. Hunter D. Smith-Warner S. A. (2004). Fruit and Vegetable Intake and Risk of Major Chronic Disease. JNCI J. Natl. Cancer Inst. 96 (21), 1577–1584. 10.1093/jnci/djh296 15523086

[B32] Jädert C. Petersson J. Massena S. Ahl D. Grapensparr L. Holm L. (2012). Decreased Leukocyte Recruitment by Inorganic Nitrate and Nitrite in Microvascular Inflammation and NSAID-Induced Intestinal Injury. Free Radic. Biol. Med. 52 (3), 683–692. 10.1016/j.freeradbiomed.2011.11.018 22178413

[B33] Jansson E. Petersson J. Reinders C. Sobko T. Bjorne H. Phillipson M. (2007). Protection from Nonsteroidal Anti-inflammatory Drug (NSAID)-induced Gastric Ulcers by Dietary Nitrate. Free Radic. Biol. Med. 42 (4), 510–518. 10.1016/j.freeradbiomed.2006.11.018 17275683

[B34] Johnson L. C. DeVan A. E. Justice J. N. Seals D. R. (2017). “Nitrate and Nitrite in Aging and Age-Related Disease,” in Nitrite and Nitrate in Human Health and Disease (Springer International Publishing AG), 259–277. 10.1007/978-3-319-46189-2_18

[B35] Jones A. M. Thompson C. Wylie L. J. Vanhatalo A. (2018). Dietary Nitrate and Physical Performance. Annu. Rev. Nutr. 38, 303–328. 10.1146/annurev-nutr-082117-051622 30130468

[B36] Justice J. N. Johnson L. C. DeVan A. E. Cruickshank-Quinn C. Reisdorph N. Bassett C. J. (2015). Improved Motor and Cognitive Performance with Sodium Nitrite Supplementation Is Related to Small Metabolite Signatures: a Pilot Trial in Middle-Aged and Older Adults. Aging 7 (11), 1004–1021. 10.18632/aging.100842 26626856 PMC4694069

[B37] Kapil V. Khambata R. S. Robertson A. Caulfield M. J. Ahluwalia A. (2015). Dietary Nitrate Provides Sustained Blood Pressure Lowering in Hypertensive Patients. Hypertension 65 (2), 320–327. 10.1161/hypertensionaha.114.04675 25421976 PMC4288952

[B38] Kelley R. C. Ferreira L. F. (2017). Diaphragm Abnormalities in Heart Failure and Aging: Mechanisms and Integration of Cardiovascular and Respiratory Pathophysiology. Heart Fail. Rev. 22 (2), 191–207. 10.1007/s10741-016-9549-4 27000754 PMC4827708

[B39] Kelley E. E. Baust J. Bonacci G. Golin-Bisello F. Devlin J. E. St. Croix C. M. (2014). Fatty Acid Nitroalkenes Ameliorate Glucose Intolerance and Pulmonary Hypertension in High-Fat Diet-Induced Obesity. Cardiovasc. Res. 101 (3), 352–363. 10.1093/cvr/cvt341 24385344 PMC3928004

[B40] Kelly J. Fulford J. Vanhatalo A. Blackwell J. R. French O. Bailey S. J. (2013). Effects of Short-Term Dietary Nitrate Supplementation on Blood Pressure, O2 Uptake Kinetics, and Muscle and Cognitive Function in Older Adults. Am. J. Physiol. Regul. Integr. Comp. Physiol. 304 (2), R73–R83. 10.1152/ajpregu.00406.2012 23174856

[B41] Kumar R. A. Kelley R. C. Hahn D. Ferreira L. F. (2020). Dietary Nitrate Supplementation Increases Diaphragm Peak Power in Old Mice. J. Physiol. 598 (19), 4357–4369. 10.1113/jp280027 33460123 PMC10195135

[B42] Larsen F. J. Ekblom B. Sahlin K. Lundberg J. O. Weitzberg E. (2006). Effects of Dietary Nitrate on Blood Pressure in Healthy Volunteers. N. Engl. J. Med. 355 (26), 2792–2793. 10.1056/nejmc062800 17192551

[B43] Larsen F. J. Schiffer T. A. Borniquel S. Sahlin K. Ekblom B. Lundberg J. O. (2011). Dietary Inorganic Nitrate Improves Mitochondrial Efficiency in Humans. Cel Metab. 13 (2), 149–159. 10.1016/j.cmet.2011.01.004 21284982

[B44] Ledo A. Barbosa R. M. Gerhardt G. A. Cadenas E. Laranjinha J. (2005). Concentration Dynamics of Nitric Oxide in Rat Hippocampal Subregions Evoked by Stimulation of the NMDA Glutamate Receptor. Proc. Natl. Acad. Sci. 102 (48), 17483–17488. 10.1073/pnas.0503624102 16293699 PMC1297656

[B45] López-Otín C. Blasco M. A. Partridge L. Serrano M. Kroemer G. (2013). The Hallmarks of Aging. Cell 153 (6), 1194–1217. 10.1016/j.cell.2013.05.039 23746838 PMC3836174

[B46] Lundberg J. O. Govoni M. (2004). Inorganic Nitrate Is a Possible Source for Systemic Generation of Nitric Oxide. Free Radic. Biol. Med. 37 (3), 395–400. 10.1016/j.freeradbiomed.2004.04.027 15223073

[B47] Lundberg J. O. Weitzberg E. (2005). NO Generation from Nitrite and its Role in Vascular Control. Arterioscler. Thromb. Vasc. Biol. 25 (5), 915–922. 10.1161/01.atv.0000161048.72004.c2 15746440

[B48] Lundberg J. O. Weitzberg E. (2013). Biology of Nitrogen Oxides in the Gastrointestinal Tract. Gut 62 (4), 616–629. 10.1136/gutjnl-2011-301649 22267589

[B49] Lundberg J. O. Weitzberg E. Lundberg J. M. Alving K. (1994). Intragastric Nitric Oxide Production in Humans: Measurements in Expelled Air. Gut 35 (11), 1543–1546. 10.1136/gut.35.11.1543 7828969 PMC1375608

[B50] Lundberg J. O. Weitzberg E. Gladwin M. T. (2008). The Nitrate-Nitrite-Nitric Oxide Pathway in Physiology and Therapeutics. Nat. Rev. Drug Discov. 7 (2), 156–167. 10.1038/nrd2466 18167491

[B51] Lundberg J. O. Gladwin M. T. Ahluwalia A. Benjamin N. Bryan N. S. Butler A. (2009). Nitrate and Nitrite in Biology, Nutrition and Therapeutics. Nat. Chem. Biol. 5 (12), 865–869. 10.1038/nchembio.260 19915529 PMC4038383

[B52] Lundberg J. O. Carlström M. Weitzberg E. (2018). Metabolic Effects of Dietary Nitrate in Health and Disease. Cel Metab. 28 (1), 9–22. 10.1016/j.cmet.2018.06.007 29972800

[B53] Millar J. (1995). The Nitric Oxide/Ascorbate Cycle: How Neurones May Control Their Own Oxygen Supply. Med. Hypotheses 45 (1), 21–26. 10.1016/0306-9877(95)90194-9 8524171

[B54] Moncada S. Higgs A. (1993). The L-Arginine-Nitric Oxide Pathway. N. Engl. J. Med. 329 (27), 2002–2012. 10.1056/NEJM199312303292706 7504210

[B55] Moncada S. Higgs E. A. (2006). The Discovery of Nitric Oxide and its Role in Vascular Biology. Br. J. Pharmacol. 147 Suppl 1 (Suppl. 1), S193–S201. 10.1038/sj.bjp.0706458 16402104 PMC1760731

[B56] Moody D. M. Brown W. R. Challa V. R. Anderson R. L. (1995). Periventricular Venous Collagenosis: Association with Leukoaraiosis. Radiology 194 (2), 469–476. 10.1148/radiology.194.2.7824728 7824728

[B57] Niccoli T. Partridge L. (2012). Ageing as a Risk Factor for Disease. Curr. Biol. 22 (17), R741–R752. 10.1016/j.cub.2012.07.024 22975005

[B58] Peleli M. Hezel M. Zollbrecht C. Persson A. E. G. Lundberg J. O. Weitzberg E. (2015). In Adenosine A2B Knockouts Acute Treatment with Inorganic Nitrate Improves Glucose Disposal, Oxidative Stress, and AMPK Signaling in the Liver. Front. Physiol. 6, 222. 10.3389/fphys.2015.00222 26300787 PMC4528163

[B59] Peleli M. Ferreira D. M. S. Tarnawski L. McCann Haworth S. Xuechen L. Zhuge Z. (2019). Dietary Nitrate Attenuates High-Fat Diet-Induced Obesity via Mechanisms Involving Higher Adipocyte Respiration and Alterations in Inflammatory Status. Redox Biol. 28, 101387. 10.1016/j.redox.2019.101387 31765889 PMC6883295

[B60] Percival R. S. Challacombe S. J. Marsh P. D. (1991). Age-related Microbiological Changes in the Salivary and Plaque Microflora of Healthy Adults. J. Med. Microbiol. 35 (1), 5–11. 10.1099/00222615-35-1-5 2072378

[B61] Pérez V. I. Bokov A. Remmen H. V. Mele J. Ran Q. Ikeno Y. (2009). Is the Oxidative Stress Theory of Aging Dead? Biochim. Biophys. Acta (Bba) - Gen. Subj. 1790 (10), 1005–1014. 10.1016/j.bbagen.2009.06.003 PMC2789432 19524016

[B62] Presley T. D. Morgan A. R. Bechtold E. Clodfelter W. Dove R. W. Jennings J. M. (2011). Acute Effect of a High Nitrate Diet on Brain Perfusion in Older Adults. Nitric Oxide 24 (1), 34–42. 10.1016/j.niox.2010.10.002 20951824 PMC3018552

[B63] Qin L. Liu X. Sun Q. Fan Z. Xia D. Ding G. (2012). Sialin (SLC17A5) Functions as a Nitrate Transporter in the Plasma Membrane. Proc. Natl. Acad. Sci. 109 (33), 13434–13439. 10.1073/pnas.1116633109 22778404 PMC3421170

[B64] Rammos C. Hendgen‐Cotta U. B. Totzeck M. Pohl J. Lüdike P. Flögel U. (2016). Impact of Dietary Nitrate on Age‐related Diastolic Dysfunction. Eur. J. Heart Fail. 18 (6), 599–610. 10.1002/ejhf.535 27118445

[B65] Raubenheimer K. Hickey D. Leveritt M. Fassett R. Ortiz de Zevallos Munoz J. Allen J. D. (2017). Acute Effects of Nitrate-Rich Beetroot Juice on Blood Pressure, Hemostasis and Vascular Inflammation Markers in Healthy Older Adults: A Randomized, Placebo-Controlled Crossover Study. Nutrients 9 (11), 1270. 10.3390/nu9111270 PMC5707742 29165355

[B66] Rocha B. S. Gago B. Barbosa R. M. Laranjinha J. (2009). Dietary Polyphenols Generate Nitric Oxide from Nitrite in the Stomach and Induce Smooth Muscle Relaxation. Toxicology 265 (1-2), 41–48. 10.1016/j.tox.2009.09.008 19778575

[B67] Rocha B. S. Gago B. Barbosa R. M. Laranjinha J. (2010). Diffusion of Nitric Oxide through the Gastric wall upon Reduction of Nitrite by Red Wine: Physiological Impact. Nitric Oxide 22 (3), 235–241. 10.1016/j.niox.2010.01.003 20083218

[B68] Rocha B. S. Gago B. Pereira C. Barbosa R. M. Bartesaghi S. Lundberg J. O. (2011). Dietary Nitrite in Nitric Oxide Biology: a Redox Interplay with Implications for Pathophysiology and Therapeutics. Curr. Drug Targets 12 (9), 1351–1363. 10.2174/138945011796150334 21443473

[B69] Rocha B. S. Gago B. Barbosa R. M. Lundberg J. O. Radi R. Laranjinha J. (2012). Intragastric Nitration by Dietary Nitrite: Implications for Modulation of Protein and Lipid Signaling. Free Radic. Biol. Med. 52 (3), 693–698. 10.1016/j.freeradbiomed.2011.11.011 22154654

[B70] Rocha B. S. Gago B. Barbosa R. M. Lundberg J. O. Mann G. E. Radi R. (2013). Pepsin Is Nitrated in the Rat Stomach, Acquiring Antiulcerogenic Activity: a Novel Interaction between Dietary Nitrate and Gut Proteins. Free Radic. Biol. Med. 58, 26–34. 10.1016/j.freeradbiomed.2012.12.017 23277149

[B71] Rocha B. S. Lundberg J. O. Radi R. Laranjinha J. (2016). Role of Nitrite, Urate and Pepsin in the Gastroprotective Effects of Saliva. Redox Biol. 8, 407–414. 10.1016/j.redox.2016.04.002 27156250 PMC4864375

[B72] Roshanravan B. Patel K. V. Fried L. F. Robinson-Cohen C. de Boer I. H. Harris T. (2017). Association of Muscle Endurance, Fatigability, and Strength with Functional Limitation and Mortality in the Health Aging and Body Composition Study. Gerona 72 (2), 284–291. 10.1093/gerona/glw210 PMC5233917 27907890

[B73] Ruitenberg A. den Heijer T. Bakker S. L. M. van Swieten J. C. Koudstaal P. J. Hofman A. (2005). Cerebral Hypoperfusion and Clinical Onset of Dementia: the Rotterdam Study. Ann. Neurol. 57 (6), 789–794. 10.1002/ana.20493 15929050

[B74] Sharma G. Goodwin J. (2006). Effect of Aging on Respiratory System Physiology and Immunology. Clin. Interventions Aging 1 (3), 253–260. 10.2147/ciia.2006.1.3.253 PMC2695176 18046878

[B75] Shiva S. Gladwin M. T. (2009). Nitrite Mediates Cytoprotection after Ischemia/reperfusion by Modulating Mitochondrial Function. Basic Res. Cardiol. 104 (2), 113–119. 10.1007/s00395-009-0009-3 19242636

[B76] Shiva S. Sack M. N. Greer J. J. Duranski M. Ringwood L. A. Burwell L. (2007). Nitrite Augments Tolerance to Ischemia/reperfusion Injury via the Modulation of Mitochondrial Electron Transfer. J. Exp. Med. 204 (9), 2089–2102. 10.1084/jem.20070198 17682069 PMC2118713

[B77] Sies H. Jones D. P. (2020). Reactive Oxygen Species (ROS) as Pleiotropic Physiological Signalling Agents. Nat. Rev. Mol. Cel Biol. 21 (7), 363–383. 10.1038/s41580-020-0230-3 32231263

[B78] Sies H. Berndt C. Jones D. P. (2017). Oxidative Stress. Annu. Rev. Biochem. 86, 715–748. 10.1146/annurev-biochem-061516-045037 28441057

[B79] Stanaway L. Rutherfurd-Markwick K. Page R. Wong M. Jirangrat W. Teh K. H. (2019). Acute Supplementation with Nitrate-Rich Beetroot Juice Causes a Greater Increase in Plasma Nitrite and Reduction in Blood Pressure of Older Compared to Younger Adults. Nutrients 11 (7), 1683. 10.3390/nu11071683 PMC6683255 31336633

[B80] Stokes K. Y. Dugas T. R. Tang Y. Garg H. Guidry E. Bryan N. S. (2009). Dietary Nitrite Prevents Hypercholesterolemic Microvascular Inflammation and Reverses Endothelial Dysfunction. Am. J. Physiol. Heart Circ. Physiol. 296 (5), H1281–H1288. 10.1152/ajpheart.01291.2008 19252084

[B81] van Faassen E. E. Bahrami S. Feelisch M. Hogg N. Kelm M. Kim-Shapiro D. B. (2009). Nitrite as Regulator of Hypoxic Signaling in Mammalian Physiology. Med. Res. Rev. 29 (5), 683–741. 10.1002/med.20151 19219851 PMC2725214

[B82] Vanhatalo A. Blackwell J. R. L’Heureux J. E. Williams D. W. Smith A. van der Giezen M. (2018). Nitrate-responsive Oral Microbiome Modulates Nitric Oxide Homeostasis and Blood Pressure in Humans. Free Radic. Biol. Med. 124, 21–30. 10.1016/j.freeradbiomed.2018.05.078 29807159 PMC6191927

[B83] Velmurugan S. Gan J. M. Rathod K. S. Khambata R. S. Ghosh S. M. Hartley A. (2016). Dietary Nitrate Improves Vascular Function in Patients with Hypercholesterolemia: a Randomized, Double-Blind, Placebo-Controlled Study. Am. J. Clin. Nutr. 103 (1), 25–38. 10.3945/ajcn.115.116244 26607938 PMC4691670

[B84] Viña J. Borrás C. Miquel J. (2007). Theories of Ageing. IUBMB Life 59 (4-5), 249–254. 10.1080/15216540601178067 17505961

[B85] Viña J. Borras C. Gomez-Cabrera M. C. (2018). A Free Radical Theory of Frailty. Free Radic. Biol. Med. 124, 358–363. 10.1016/j.freeradbiomed.2018.06.028 29958933

[B86] Wan S.-H. Vogel M. W. Chen H. H. (2014). Pre-Clinical Diastolic Dysfunction. J. Am. Coll. Cardiol. 63 (5), 407–416. 10.1016/j.jacc.2013.10.063 24291270 PMC3934927

[B87] Wang H. Hu L. Li L. Wu X. Fan Z. Zhang C. (2018). Inorganic Nitrate Alleviates the Senescence-Related Decline in Liver Function. Sci. China Life Sci. 61 (1), 24–34. 10.1007/s11427-017-9207-x 29307111

[B88] Webb A. Bond R. McLean P. Uppal R. Benjamin N. Ahluwalia A. (2004). Reduction of Nitrite to Nitric Oxide during Ischemia Protects against Myocardial Ischemia-Reperfusion Damage. Proc. Natl. Acad. Sci. 101 (37), 13683–13688. 10.1073/pnas.0402927101 15347817 PMC518813

[B89] Webb A. J. Patel N. Loukogeorgakis S. Okorie M. Aboud Z. Misra S. (2008). Acute Blood Pressure Lowering, Vasoprotective, and Antiplatelet Properties of Dietary Nitrate via Bioconversion to Nitrite. Hypertension 51 (3), 784–790. 10.1161/hypertensionaha.107.103523 18250365 PMC2839282

[B90] Weitzberg E. Lundberg J. O. (2013). Novel Aspects of Dietary Nitrate and Human Health. Annu. Rev. Nutr. 33, 129–159. 10.1146/annurev-nutr-071812-161159 23642194

[B91] Wightman E. L. Haskell-Ramsay C. F. Thompson K. G. Blackwell J. R. Winyard P. G. Forster J. (2015). Dietary Nitrate Modulates Cerebral Blood Flow Parameters and Cognitive Performance in Humans: A Double-Blind, Placebo-Controlled, Crossover Investigation. Physiol. Behav. 149, 149–158. 10.1016/j.physbeh.2015.05.035 26037632

